# Controlled bidirectional teleportation of unknown single-particle states by using an arbitrary high-dimensional entangled state

**DOI:** 10.1371/journal.pone.0332405

**Published:** 2025-09-15

**Authors:** Huangrui Lei, Jiangang Tang, Jiayin Peng

**Affiliations:** 1 Department of Mathematics, Sichuan University Jinjiang College, Meishan, Sichuan, China; 2 School of Mathematics and Statistics, Yili Normal University, Yining, Xinjiang, China; 3 School of Mathematics and Information Science, Neijiang Normal University, Neijiang, Sichuan, China; Universite Cote d’Azur, FRANCE

## Abstract

This study investigates controlled bidirectional transmission of two low-dimensional unknown single-particle quantum states through high-dimensional entangled channels. Initially, complete orthogonal non-symmetric bases in (d×f)-dimensional Hilbert space and five-particle maximally entangled states in *d*-dimensional Hilbert space are constructed, with (3×2)-dimensional non-symmetric bases and 3-dimensional 5-qutrit entangled states serving as specific instances. We then propose a controlled bidirectional teleportation (CBT) protocol wherein a 3-dimensional 5-qutrit maximally entangled channel enables simultaneous exchange of two 2-dimensional single-qubit states under supervisor authorization, implemented via non-symmetric basis measurements. Subsequent replacement of the maximally entangled channel with a 3-dimensional 5-qutrit non-maximally entangled state facilitates probabilistic reconstruction of the original states through auxiliary particle introduction and appropriate unitary operations, still under supervisory control. The success probabilities are analytically derived, demonstrating that the non-maximally entangled protocol generalizes the former scheme. Furthermore, these protocols admit dual extensions: (i) quantum channel dimensionality scalable from 3-dimensional to arbitrary *d* dimensions; (ii) transmitted state dimensionality extendable from 2-dimensional to arbitrary *f*-dimensional Hilbert space (*f* < *d*).

## 1 Introduction

Quantum information is a new and cross-disciplinary science based mainly on the principles of quantum mechanics, and quantum teleportation (QT) is one of many interesting and striking protocols in the field which has received extensive attention since its inception. This technique allows two remote parties to exploit the nonlocal correlations of an initially shared quantum channel for the disembodied transport of quantum state from a place to another one through a classical communication channel prearranging the share of resource of entanglement. The original QT protocol of Bennett et al. [1] teleports an unknown qubit from sender to a spatially separated receiver using an Einstein-Podolsky-Rosen (EPR) pair and by sending two bit of classical information from sender to receiver. D. Bouwmeester et al. [2] later proposed how to execute experimentation on QT with the polarization photon. In 1998, A. Furusawa et al. [3] demonstrated QT with a single coherent mode of field in optical experiment. Thereafter, some experiments have also demonstrated QT of a single qubit with entangled photons and ions [4–8]. Currently, the majority of theoretical protocols for QT have been considered with different quantum channels [9–20]. In 2002, Karlsson and Bourennane [9] suggested a controlled quantum teleportation (CQT) scheme using three-particle entanglement, allowing the sender to transfer quantum information to the receiver with a third party serving as the controller. Lu and Gao [10] presented some protocols for transmitting a two-particle quantum system in a Bell-class entangled state. Lee [11] put forward a scheme for transmitting a two-qubit entangled state by using the four-qubit GHZ state. Yan and Yang [12] presented an economic QT protocol of multi-qubit state which uses only one EPR pair to teleport an unknown GHZ-class state. Zuo et al. [13] discussed the quantum splitting or quantum teleportation of single-qubit state with *W* state as quantum channel. Gao proposed controlled QT schemes with several kinds of three-qubit states. In 2021, Yu et al. [15] introduced the multi-output QT scheme to symmetrically transmit the arbitrary *m*- and (m+1)-qubit GHZ-class states from one sender to two receivers via a five-qubit entangled state. Peng et al. [16] studied correlated effects of Pauli Noise on controlled QT of an arbitrary single-subit state through a three-qubit W state. Literature [17] pointed out that perfect QT is suitable with Star type tripartite states, and also standard QT is possible by using the linear superposition of non-prototype W state and its spinflipped version. In 2024, Peng et al. [18] proposed new scheme for QT between multiple senders and receivers, which allows the high-dimensional quantum information shared by an arbitrary number of senders to transferred to another arbitrary number of receivers.

Nevertheless, in the QT scheme recalled above, only one transmission direction was involved. The concept of bidirectional teleportation (BT) emerged in 2001 [19], which allows two users to simultaneously exchange their respective quantum states with each other. This breakthrough invention has attracted widespread attention from many researchers [20–25]. Expanding on the idea of Karlsson et al. [7], Zha et al. [26] put forward a bidirectional controlled QT (BCQT) scheme via a five-qubit cluster state as quantum channel. This allows two users to serve as both sender and receiver, exchanging their respective arbitrary unknown single-qubit states with each other under the control of the third user acting as a supervisor. Later, various schemes for BQT and BCQT using the entangled multi-qubit as quantum channel have been proposed in reference [27–41]. Most schemes of BQT or BCQT establish various teleportation procedures, such as increasing the number of transmitted qubit states or reducing the number of qubits in the quantum channel [27–34], multi controlling communication to improve connection security [35], and asymmetric CQT, where the number of qubit states in sender and receiver are different [36–41]. Some other schemes of two-way quantum communication use hybrid communications, where CQT appears together with the remote state preparation (RSP) scheme and prepares a state for one of parties [42–44]. Reference [45] employed entanglement swapping. In [46–49], cyclic QT and CQT were studied, where Alice transmits a single-qubit state to Bob and Bob teleports a single-qubit state to Charlie, at the same time, Charlie also teleports a single-qubit state back to Alice. In the study [50], any one of the three users can play the role of a supervisor. Some recent developments have proposed universal schemes for QT of *n*-arbitrary qubit states [51,52]. Zhou et al. [53,54] suggested several schemes in which the 3-qubit entangled states can be transformed into 2-qubit entangled states and single superposition qubit state using Toffoli and CNOT operators, and then the initial qubits can be reconstructed at the receiver position. Chen et al. [55] presented a bidirectional QT scheme for mutually teleporting two single-qubit state and an unknown three-qubit state by using a four-qubit GHZ state and two Bell states as the quantum channel. Meanwhile, some scholars have proposed the schemes for probabilistic teleportation. Peng et al. [20] suggested two bidirectional QT schemes in amplitude damping channels to respectively exchange two arbitrary single-qubit states and two arbitrary single-qutrit states between two users. Yan et al. [56] put forward a scheme for probabilistic and controlled teleportation of the unknown quantum states of both one-particle and two-particle. Ref. [57] presents a scheme for probabilistic teleportation of an arbitrary GHZ-class state with a pure entangled two-particle quantum channel.

However, in all of the above schemes, the dimension, in the Hilbert space, of particle(s) of quantum state sender wishes to teleport is the same as that of the quantum channel shared by sender and receiver. In 2001, Zhou and Hou [58] introduced QT scheme where unknown quantum pure state of an *S*-level particle can be transferred onto a group of remote two-level particles with the aid of two-level Einstein-Podolsky-Rosen states. Ten years later, Zhan et al. [59] proposed a scheme to teleport an unknown quantum state of a *f*-dimensional single particle by using a *d*-dimensional two-particle entangled state. Obviously, in these two paper, the dimension of the Hilbert space for the particle of the state to be transferred lie are different from that of the Hilbert space in which the particles corresponding to the quantum channel lie. In the former article, the Hilbert space dimension of the transmitted particle is greater than the Hilbert space dimension of the particle in the quantum channel, and in the latter article the reverse is true. That is, in these two articles, quantum information occurs on the transfer of particles of different dimensions between two parties, quantum state transmission is a very simple one-way transfer, and all quantum channels are in maximally entangled state(s). One naturally wonders what would happen if the transmission of unknown single-particle states is bidirectional, controlled, and the state used as a quantum channel is some non-maximally entangled multi-particle state? To our knowledge, there is currently no solution to this problem. [60] Due to the stronger robustness of high-dimensional quantum entangled states to noise compared to low dimensional quantum entangled states, and the higher security of controlled schemes, bidirectional schemes can lead to flexible and diverse communication. Therefore, studying controlled bidirectional teleportation (CBT) using high-dimensional entangled states as quantum channels has theoretical value and practical significance. Inspired by the above problem, and based on [59], the aim of this paper is to investigate the CBT of low-dimensional single-particle entangled states via a high-dimensional entangled states acting as quantum channel. To this end, we first construct the non-symmetric basis and high-dimensional maximally entangled 5-particle state, based on them, present a scheme for CBT of unknown single-qubit states through a three-dimensional maximally entangled 5-qutrit state as the quantum channel. Then, consider the CBT scheme in the case of a three-dimensional non-maximally entangled 5-qutrit state as quantum channel. In addition, two types of extensions of the above schemes are considered: the extension of quantum channels and the transmitted quantum states to arbitrary high-dimensional situation (the dimension of the former is greater than that of the latter). For other quantum channel distributions, the methodology is similar and thus will not be repeated. The controller introduced in our scheme provides an additional security barrier for communication.

The arrangement of this article is as follows: in Section 2 we mainly construct the (3×2)-, (d×2)- and (d×f)-dimensional non-symmetric measurement bases, and then construct 3-dimensional and *d*-dimensional maximally entangled 5-particle states. In Section 3, we present a CBT protocol to exchange two unknown single-qubit entangled states by using a three-dimensional maximally entangled 5-qutrit state, and then extend it to the case of a three-dimensional non-maximally entangled channel. In Sections 4, we generalized two schemes in Section 3 to the case of a *d*-dimensional maximally or non-maximally entangled 5-qudit state as quantum channel. In Section 5, we extend the transmitted 2-dimensional single-qubit states of the schemes in Section 4 to the general high-dimensional case. Finally, a brief discussion is conducted in Section 6 and conclusions are drawn.

## 2 Preliminary and working channel

### 2.1 Preliminary knowledge

In this subsection we briefly review some basic concepts and terminologies [59], and based on them we present the construction procedure of the quantum channels needed in our controlled bidirectional teleportation (CBT) schemes in following.

A arbitrary single-qudit state in *d*-dimensional Hilbert space can be written as a complex linear combination in the standard basis {|j⟩} with j=1,2,⋯,d−1:

$$|\varphi \rangle =\sum_{j=0}^{d-1}\alpha_{j}|j\rangle ,$$ (1)

where all complex numbers $\alpha_{j}$ satisfies the normalization condition $\sum_{j=0}^{d-1}|\alpha_{j}{|}^{2}=1$ and d≥2. Bell-like Basis $\left\{|{ℬ}_{mn}\rangle |:m,n=0,1,\cdots d\;-\;1\right\}$ (Simply represent it as $\left\{|{ℬ}_{mn}\rangle \right\}$), with elements given by

$$|{ℬ}_{mn}\rangle =\sum_{j=0}^{d-1}\zeta_{jm}|j,(j+n)modd\rangle ,$$ (2)

where the $\zeta_{jm}$ coefficients account for the extent of entanglement and satisfy the relation $\sum_{j=0}^{d-1}\zeta_{jm}\zeta_{j^{\prime }m^{\prime }}^{*}=\delta_{jj^{\prime }}\delta mm^{\prime }$ (orthonormality) and $tr_{2}(|{ℬ}_{mn}\rangle \langle {ℬ}_{mn}|)=d^{-1}I$ (maximal entanglement), here $\zeta_{jm}=\omega_{d}^{jm}/\sqrt{d}$, while $\omega_{d}=\exp(2\pi i/d)$ is the primitive *d*th root of unity. The following operators [38–40], which are also called Weyl operators, are also very useful, since they play the analogous role of Pauli operators for qudits:

$$U_{mn}=\sum_{j=0}^{d-1}\omega_{d}^{jm}|j\rangle \langle (j+n)modd\rangle ,\;\;m,n=0,1,\cdots ,d-1,$$ (3)

which satisfy $(I\;⊗\;U_{mn})|{ℬ}_{00}\rangle =|{ℬ}_{mn}\rangle$. One should, however, note that contrary to the Pauli operators, the operators *U*_*mn*_ are not necessarily Hermitian.

One can also generalize the Hadamard gate that turns out to be quite useful in manipulating qudits for various applications [39,40],

$$H_{d}=\sum_{j,k=0}^{d-1}\zeta_{jk}|j\rangle \langle k|.$$ (4)

The operator is really not new and it is known as the quantum Fourier transform when *d* = 2^*n*^. In that case it acts on *n* qubits. Here we are assuming it to be a basic gate on one single qudit, in the same way as the ordinary Hadamard gate is a basic gate on one qubit. The operator is symmetric and unitary ($H_{d}H_{d}^{*}=I$), but not Hermitian.

To generalize the NOT and the controlled NOT (CNOT) gates, we note that in the context of qudits, the NOT gate is, basically, a mod-2 adder. For qudits this operator gives way to a mod-*d* adder [39,40],

$${𝒩}_{d}^{\left(A,B\right)}|i\rangle_{A}|j\rangle_{B}=|i\rangle_{A}|(i+j)modd\rangle_{B},$$ (5)

$${\tilde{N}}_{d}^{\left(A,B\right)}|i\rangle_{A}|j\rangle_{B}=|i\rangle_{A}|(i-j)modd\rangle_{B},$$ (6)

where ${𝒩}_{d}^{\left(A,B\right)}$ and ${\tilde{N}}_{d}^{\left(A,B\right)}$ are the *d*-dimensional CNOT gate and the *d*-dimensional inverse CNOT gate, respectively, where particle *A* is the control particle, and particle *B* is the target particle.

We introduce the maximally entangled state in (3×2)-dimensional Hilbert space [36,37]

$$|\Phi_{00}\rangle_{CD}=\frac{1}{\sqrt{2}}(|00\rangle +|11\rangle {)}_{CD},$$ (7)

where particle *C* is in 3-dimensional Hilbert space and particle *D* is in 2-dimensional Hilbert space. The single-body operations

$$U_{st}=|s\rangle \langle 0|+(-1{)}^{t}|(s+1)mod3\rangle \langle 1|+|(s+2)mod3\rangle \langle 2|,$$ (8)

where s∈{0,1,2} and t∈{0,1}, on particle *C* will transform non-symmetric entangled state $|\Phi_{00}\rangle_{CD}$ into the states

$$U_{st}|\Phi_{00}\rangle_{CD}=|\Phi_{st}\rangle_{CD},$$ (9)

where $|\Phi_{st}\rangle_{CD}=\frac{1}{\sqrt{2}}[|s,0\rangle +(-1{)}^{t}|(s+1)mod3,1\rangle {]}_{CD}$. Explicitly, the single-body operations

$$U_{00}=|0\rangle \langle 0|+|1\rangle \langle 1|+|2\rangle \langle 2|,\;U_{01}=|0\rangle \langle 0|-|1\rangle \langle 1|+|2\rangle \langle 2|,\;U_{10}=|1\rangle \langle 0|+|2\rangle \langle 1|+|0\rangle \langle 2|,\;U_{11}=|1\rangle \langle 0|-|2\rangle \langle 1|+|0\rangle \langle 2|,\;U_{20}=|2\rangle \langle 0|+|0\rangle \langle 1|+|1\rangle \langle 2|,\;U_{21}=|2\rangle \langle 0|-|0\rangle \langle 1|+|1\rangle \langle 2|,\;$$ (10)

on particle *C* will transform non-symmetric entangled state $|\Phi_{00}\rangle_{CD}$ into the corresponding states, respectively

$$U_{00}|\Phi_{00}\rangle_{CD}=\frac{1}{\sqrt{2}}(|00\rangle +|11\rangle {)}_{CD}=|\Phi_{00}\rangle_{CD},\;U_{01}|\Phi_{00}\rangle_{CD}=\frac{1}{\sqrt{2}}(|00\rangle -|11\rangle {)}_{CD}=|\Phi_{01}\rangle_{CD},\;U_{10}|\Phi_{00}\rangle_{CD}=\frac{1}{\sqrt{2}}(|10\rangle +|21\rangle {)}_{CD}=|\Phi_{10}\rangle_{CD},\;U_{11}|\Phi_{00}\rangle_{CD}=\frac{1}{\sqrt{2}}(|10\rangle -|21\rangle {)}_{CD}=|\Phi_{11}\rangle_{CD},\;U_{20}|\Phi_{00}\rangle_{CD}=\frac{1}{\sqrt{2}}(|20\rangle +|01\rangle {)}_{CD}=|\Phi_{20}\rangle_{CD},\;U_{21}|\Phi_{00}\rangle_{CD}=\frac{1}{\sqrt{2}}(|20\rangle -|01\rangle {)}_{CD}=|\Phi_{21}\rangle_{CD}.\;$$ (11)

These six states $|\Phi_{st}\rangle$ (s=0,1,2; t=0,1) form an orthogonal and complete basis $\left\{|\Phi_{st}\rangle \right\}$, i.e., $\sum_{s}\sum_{t}|\Phi_{st}\rangle \langle \Phi_{st}|=I$, $\langle \Phi_{st}|\Phi_{s^{\prime }t^{\prime }}\rangle =\delta_{ss^{\prime }}\delta_{tt^{\prime }}$ ($s^{\prime }=0,1,2;t^{\prime }=0,1$), here *I* is the identity operator.

The above concepts can be directly generalized to the high-dimensional Hilbert space. Consider the maximally entangled state in (d×2)-dimensional Hilbert space [36-37]

$$|\Psi_{00}\rangle_{CD}=\frac{1}{\sqrt{2}}(|00\rangle +|11\rangle {)}_{CD},$$ (12)

where particle *C* is in *d*-dimensional Hilbert space and particle *D* is in 2-dimensional Hilbert space, and the single-body unitary operation

$${𝒰}_{kl}=\sum_{j=0}^{d-1}(-1{)}^{lj}|(j+k)modd\rangle \langle j|,$$ (13)

where k=0,1,⋯,d−1; l=0,1. Specifically,

$${𝒰}_{00}=|0\rangle \langle 0|+|1\rangle \langle 1|+\cdots +|d-1\rangle \langle d-1|,\;{𝒰}_{01}=|0\rangle \langle 0|-|1\rangle \langle 1|+\cdots +(-1{)}^{d-1}|d-1\rangle \langle d-1|,\;{𝒰}_{10}=|1\rangle \langle 0|+|2\rangle \langle 1|+\cdots +|0\rangle \langle d-1|,\;\vdots \;{𝒰}_{h0}=|h\rangle \langle 0|+|h+1\rangle \langle 1|+\cdots +|h-1\rangle \langle d-1|,\;{𝒰}_{h1}=|h\rangle \langle 0|-|h+1\rangle \langle 1|+\cdots +(-1{)}^{d-1}|h-1\rangle \langle d-1|,\;\vdots \;{𝒰}_{d-1,0}=|d-1\rangle \langle 0|+|0\rangle \langle 1|+\cdots +|d-2\rangle \langle d-1|,\;{𝒰}_{d-1,1}=|d-1\rangle \langle 0|-|0\rangle \langle 1|+\cdots +(-1{)}^{d-1}|d-2\rangle \langle d-1|,\;$$ (14)

where h≤d−1. The single-body unitary operation ${𝒰}_{kl}$ on particle *C* will transform $|\Psi_{00}\rangle_{CD}$ into the corresponding state ${𝒰}_{kl}|\Psi_{00}\rangle_{CD}=|\Psi_{kl}\rangle_{CD}$. Concretely,

$${𝒰}_{00}|\Psi_{00}\rangle_{CD}=\frac{1}{\sqrt{2}}(|00\rangle +|11\rangle {)}_{CD}=|\Psi_{00}\rangle_{CD},\;{𝒰}_{01}|\Psi_{00}\rangle_{CD}=\frac{1}{\sqrt{2}}(|00\rangle -|11\rangle {)}_{CD}=|\Psi_{01}\rangle_{CD},\;{𝒰}_{10}|\Psi_{00}\rangle_{CD}=\frac{1}{\sqrt{2}}(|10\rangle +|21\rangle {)}_{CD}=|\Psi_{10}\rangle_{CD},\;\vdots \;{𝒰}_{h0}|\Psi_{00}\rangle_{CD}=\frac{1}{\sqrt{2}}(|h0\rangle +|h+1,1\rangle {)}_{CD}=|\Psi_{h0}\rangle_{CD},\;{𝒰}_{h1}|\Psi_{00}\rangle_{CD}=\frac{1}{\sqrt{2}}(|h0\rangle -|h+1,1\rangle {)}_{CD}=|\Psi_{h1}\rangle_{CD},\;\vdots \;{𝒰}_{d-1,0}|\Psi_{00}\rangle_{CD}=\frac{1}{\sqrt{2}}(|d-1,0\rangle +|01\rangle {)}_{CD}=|\Psi_{d-1,0}\rangle_{CD},\;{𝒰}_{d-1,1}|\Psi_{00}\rangle_{CD}=\frac{1}{\sqrt{2}}(|d-1,0\rangle -|01\rangle {)}_{CD}=|\Psi_{d-1,1}\rangle_{CD}.\;$$ (15)

These 2*d* states $|\Psi_{kl}\rangle$ (k=0,1,⋯,d−1;l=0,1) form a orthogonal and complete basis $\left\{|\Psi_{kl}\rangle \right\}$, i.e., $\sum_{k}\sum_{l}|\Psi_{kl}\rangle \langle \Psi_{kl}|=I$, $\langle \Psi_{kl}|\Psi_{k^{\prime }l^{\prime }}\rangle =\delta_{kk^{\prime }}\delta_{ll^{\prime }}$ ($k^{\prime }=0,1,\cdots ,d-1;l^{\prime }=0,1$), here *I* is the identity operator.

More generally, we can generalize the above non-symmetric basis to the more general case, which is composed vectors of two particles of arbitrary different dimensions. Consider a maximally entangled state in (d×f)-dimensional Hilbert space

$$|\Upsilon_{00}\rangle_{xy}=\frac{1}{\sqrt{f}}(|00\rangle +|11\rangle +\cdots +|f-1,f-1\rangle {)}_{xy},$$ (16)

where particle *x* is in *d*-dimensional Hilbert space and particle *y* is in *f*-dimensional Hilbert space, and *f*<*d*. The single-body unitary operation ${\hat{U}}_{st}$

$${\hat{U}}_{st}=\sum_{j=0}^{f-1}e^{2\pi itj/f}|(j+s)modd\rangle \langle j|+\sum_{j=f}^{d-1}|(j+s)modd\rangle \langle j|,$$ (17)

on particle *x* will transform $|\Upsilon_{00}\rangle$ into the corresponding state ${\hat{U}}_{st}|\Upsilon_{00}\rangle =|\Upsilon_{st}\rangle$, i.e.,

$${\hat{U}}_{st}|\Upsilon_{00}\rangle =\frac{1}{\sqrt{f}}\sum_{j=0}^{f-1}e^{2\pi itj/f}|(j+s)modd\rangle |j\rangle =|\Upsilon_{st}\rangle ,$$ (18)

where s∈{0,1,⋯,d−1},t∈{0,1,⋯,f−1}. These *df* states $|\Upsilon_{st}\rangle$ (s=0,1,⋯,d−1;t=0,1,⋯,f−1) form a orthogonal and complete basis $\left\{|\Upsilon_{st}\rangle \right\}$, i.e., $\sum_{s}\sum_{t}|\Upsilon_{st}\rangle \langle \Upsilon_{st}|=I$, $\langle \Upsilon_{st}|\Upsilon_{s^{\prime }t^{\prime }}\rangle =\delta_{ss^{\prime }}\delta_{tt^{\prime }}$ ($s^{\prime }=0,1,\cdots ,d-1;t^{\prime }=0,1,\cdots ,f-1$), here *I* is the identity operator.

### 2.2 The construction of the working channel

Now, we are going to construct several quantum states which will work as the quantum channels of the new schemes proposed in this paper, respectively. Firstly, we construct a 3-dimensional maximally entangled 5-qutrit channel $|{𝒞}_{3}\rangle$ [61] as follows

$$|{𝒞}_{3}\rangle =\frac{1}{3\sqrt{3}}\sum_{j=0}^{2}|j\rangle_{1}\sum_{l=0}^{2}e^{2\pi ijl/3}|ll\rangle_{23}\sum_{l=0}^{2}e^{2\pi ijl/3}|ll\rangle_{45}.$$ (19)

The specific construction process is as follows: the input state is a five-qutrit product state $|00000\rangle_{12345}$, and the qutrit 1 is fed into a 3-dimensional Hadamard gate *H*_3_, which transforms the initial input into

$$|\varphi_{1}\rangle =(H_{3}\;⊗\;I\;⊗\;I\;⊗\;I\;⊗\;I)|00000\rangle_{12345}=\frac{1}{\sqrt{3}}\sum_{j=0}^{2}|j\rangle_{1}|0000\rangle_{2345},$$ (20)

and then sends qutrits 2 and 4 to 3-dimensional controlled NOT gate ${𝒩}_{3}$, where qutrit 1 acts as control qutrit, and qutrits 2 and 4 as target qutrits, we have

$$|\varphi_{2}\rangle =({𝒩}_{3}^{\left(1,4\right)}\;⊗\;I_{235}^{\;⊗\;3})[({𝒩}_{3}^{\left(1,2\right)}\;⊗\;I_{345}^{\;⊗\;3})|\varphi_{1}\rangle ]=\frac{1}{\sqrt{3}}\sum_{j=0}^{2}|j\rangle_{1}|j,0,j,0\rangle_{2345},$$ (21)

where $I_{235}^{\;⊗\;3}$ represents the identity transformation on particles 1, 3 and 5, and $I_{345}^{\;⊗\;3}$ represents the identity transformation on particles 3, 4 and 5. Now, we preform two 3-dimensional Hadamard gates on qutrits 2 and 4, i.e.,

$$|\varphi_{3}\rangle =(I\;⊗\;H_{3}\;⊗\;I\;⊗\;H_{3}\;⊗\;I)|\varphi_{2}\rangle \;=\frac{1}{3\sqrt{3}}\sum_{j=0}^{2}|j\rangle_{1}\sum_{l=0}^{2}e^{2\pi ijl/3}|l0\rangle_{23}\sum_{l=0}^{2}e^{2\pi ijl/3}|l0\rangle_{45}.$$ (22)

Finally, we perform two 3-dimensional controlled NOT gates on qutrits 3 and 5, where qutrits 2 and 4 act as control qutrits, and qutrits 3 and 5 as target qutrits, respectively, which evolve the state $|\varphi_{3}\rangle$ to

$$\;\;(I_{1}\;⊗\;{𝒩}_{3}^{\left(2,3\right)}\;⊗\;{𝒩}_{3}^{\left(4,5\right)})|\varphi_{3}\rangle \;=\frac{1}{3\sqrt{3}}\sum_{j=0}^{2}|j\rangle_{1}\sum_{l=0}^{2}e^{2\pi ijl/3}|ll\rangle_{23}\sum_{l=0}^{2}e^{2\pi ijl/3}|ll\rangle_{45},$$ (23)

where *I*_1_ represents the identity transformation on particle 1.

Obviously, $|{𝒞}_{3}\rangle =(I\;⊗\;{𝒩}_{3}\;⊗\;{𝒩}_{3})|\varphi_{3}\rangle$, completing the construction.

Similarly, we can also construct the following *d*-dimensional maximally entangled five-particle state

$$|{𝒞}_{d}\rangle =\frac{1}{d\sqrt{d}}\sum_{j=0}^{d-1}|j\rangle_{1}\sum_{l=0}^{d-1}e^{2\pi ijl/d}|ll\rangle_{23}\sum_{l=0}^{2}e^{2\pi ijl/d}|ll\rangle_{45}.$$ (24)

## 3 CBT of unknown single-qubit states via a 3-dimensional entangled state

### 3.1 CBT based on a 3-dimensional maximally entangled state

Assuming Alice, Bob and Charlie are three spatially separated legitimate participants, and they preshare a 3-dimensional maximally entangled 5-particle state $|{𝒞}_{3}\rangle$ as shown Eq (19) in such way that qutrit 1 belongs to Charlie, qutrits 2 and 4 belong to Alice, and qutrits 3 and 5 belong to Bob. Suppose that Alice has an unknown single-qubit state

$$|\xi \rangle_{A}=(\alpha_{0}|0\rangle +\alpha_{1}|1\rangle {)}_{A},$$ (25)

where $\alpha_{0}$ and $\alpha_{1}$ are complex numbers with $\sum_{j=0}^{1}|\alpha_{j}{|}^{2}=1$, She wants to teleport this state to Bob, at the same time, Bob has also an unknown single-qubit state

$$|\eta \rangle_{B}=(\beta_{0}|0\rangle +\beta_{1}|1\rangle {)}_{B},$$ (26)

where $\beta_{0}$ and $\beta_{1}$ are complex numbers with $\sum_{j=0}^{1}|\beta_{j}{|}^{2}=1$, he intends to teleport the state $|\eta \rangle_{B}$ to Alice under the control of the supervisor Charlie.

The state of the whole initial system may be expressed as

$$|G\rangle =|\xi \rangle_{A}⊗|\eta \rangle_{B}⊗|{𝒞}_{3}\rangle \;=\frac{1}{3\sqrt{3}}(\alpha_{0}|0\rangle +\alpha_{1}|1\rangle {)}_{A}⊗(\beta_{0}|0\rangle +\beta_{1}|1\rangle {)}_{B}\;\;\;⊗\sum_{j=0}^{2}|j\rangle_{1}\sum_{l=0}^{2}e^{2\pi ijl/3}|ll\rangle_{23}\sum_{l=0}^{2}e^{2\pi ijl/3}|ll\rangle_{45}.\;=\frac{1}{6\sqrt{3}}\sum_{j=0}^{2}|j\rangle_{1}\{[\sum_{s=0}^{2}\sum_{t=0}^{1}|\Phi_{st}\rangle_{2A}\sum_{l=0}^{1}(-1{)}^{tl}\exp\{\frac{2\pi i}{3}j(s+l)mod3\}\alpha_{l}|(s+l)mod3\rangle_{3}]\;\;\;⊗[\sum_{m=0}^{2}\sum_{n=0}^{1}|\Phi_{mn}\rangle_{5B}\sum_{k=0}^{1}(-1{)}^{nk}\exp\{\frac{2\pi i}{3}j(m+k)mod3\}\beta_{k}|(m+k)mod3\rangle_{4}]\}.$$ (27)

To complete the quantum task, Alice needs to measure her particle pair (2,*A*) with the non-symmetric basis $\left\{|\Phi_{st}\rangle \right\}$ as shown in Eq (11), and then tells Bob of her measurement result $|\Phi_{st}\rangle_{2A}$ through a classical channel. At the same time, Bob also performs a non-symmetric basis $\left\{|\Phi_{st}\rangle \right\}$ measurement on his particle pair (5,*B*), and informs Alice of his measurement result $|\Phi_{mn}\rangle_{5B}$ through a classical channel.

After performing the measurements, for the generic measurement outcomes $|\Phi_{st}\rangle_{2A}$ and $|\Phi_{mn}\rangle_{5B}$ (s,m∈{0,1,2} and t,n∈{0,1}), the state of the remaining qutrits 1, 3 and 4 collapses into

$$|G^{\prime }\rangle =\frac{1}{\sqrt{3}}\sum_{j=0}^{2}|j\rangle_{1}\{[\sum_{l=0}^{1}(-1{)}^{tl}\exp\{\frac{2\pi i}{3}j(s+l)mod3\}\alpha_{l}|(s+l)mod3\rangle_{3}]\;\;\;⊗[\sum_{k=0}^{1}(-1{)}^{nk}\exp\{\frac{2\pi i}{3}j(m+k)mod3\}\beta_{k}|(m+k)mod3\rangle_{4}]\}.$$ (28)

If the supervisor Charlie is willing to cooperate, he needs to perform a single-qutrit projective measurement on his qutrit 1 with *Z*-basis {|0⟩,|1⟩,|2⟩}, and tells his measurement outcome $|h\rangle_{1}$ (h=0,1,2) to Alice and Bob via the classical channels. After Charlie measurement, the state of qutrits 3 and 4 will collapse into

$$|G^{\prime \prime }\rangle =[\sum_{l=0}^{1}(-1{)}^{tl}\exp\{\frac{2\pi i}{3}h(s+l)mod3\}\alpha_{l}|(s+l)mod3\rangle_{3}]\;\;\;⊗[\sum_{k=0}^{1}(-1{)}^{nk}\exp\{\frac{2\pi i}{3}h(m+k)mod3\}\beta_{k}|(m+k)mod3\rangle_{4}].$$ (29)

According to the measurement outcomes from Bob and Charlie, Alice can perform a unitary operation *U*_*mnh*_ on qutrit 4, which is given by

$$U_{mnh}=\sum_{k=0}^{1}(-1{)}^{nk}\exp\{\frac{-2\pi i}{3}h(m+k)mod3\}|k\rangle \langle (m+k)mod3|.$$ (30)

At the same time, Bob also executes a unitary operation *U*_*sth*_ on qutrit 3, which is given by

$$U_{sth}=\sum_{l=0}^{1}(-1{)}^{tl}\exp\{\frac{-2\pi i}{3}h(s+l)mod3\}|l\rangle \langle (s+l)mod3|,$$ (31)

after hearing the measurement information form Alice and Charlie. That is,

$$(U_{sth}\;⊗\;U_{mnh})|G^{\prime \prime }\rangle =[\sum_{l=0}^{1}\alpha_{l}|l\rangle {]}_{3}⊗[\sum_{k=0}^{1}\beta_{k}|k\rangle {]}_{4},$$ (32)

which means that Alice and Bob successfully reconstruct the target states |η⟩ and |ξ⟩ on their qutrits 4 and 3, respectively. In other words, Alice and Bob successfully exchange their quantum states under the control of the supervisor Charlie.

### 3.2 CBT based on a 3-dimensional non-maximally entangled state

Now, let us compute the probability that our scheme succeeds. From Eq (27), Alice obtains the results $|\Phi_{st}\rangle_{2A}$, Bob gets the result $|\Phi_{mn}\rangle_{5B}$ and Charlie obtains the outcome $|h\rangle_{1}$ with the probability of $(1/6\sqrt{3}{)}^{2}=1/108$. Since s,m,h∈{0,1,2} and t,n∈{0,1}, the measurements results produced by the participants are in total 3×3×3×2×2=108 groups. From Eqs (29) to (32), it is known that any of their sets of measurement results can successfully exchange quantum information with the probability of 1/108. Thus, the overall success probability of our scheme is 1/108×108=1.

In fact, it is possible to teleport via a 3-dimensional non-maximally entangled state as the quantum channel. Suppose the quantum states that Alice and Bob want to exchange are still $|\xi \rangle_{A}$ and $|\eta \rangle_{B}$ as shown in Eqs (25) and (26). The entangled qutrit group shared by Alice, Bob and Charlie is a 3-dimsional non-maximally entangled 5-qutrit state, which is given by

$$|{𝒞}_{3}^{\prime }\rangle =\frac{1}{\sqrt{3}}\sum_{j=0}^{2}|j\rangle_{1}\sum_{l=0}^{2}e^{2\pi ijl/3}a_{l}|ll\rangle_{23}\sum_{l=0}^{2}e^{2\pi ijl/3}b_{l}|ll\rangle_{45}.$$ (33)

where $a_{0},a_{1},a_{2},b_{0},b_{1}$ and *b*_2_ are real numbers and satisfy the normalization conditions $\sum_{j=0}^{3}a_{j}^{2}=1$ and $\sum_{j=0}^{3}b_{j}^{2}=1$. Qutrit 1 belongs to Charlie, qutrits 2 and 4 belong to Alice, and qutrits 3 and 5 belong to Bob, respectively. The entire initial system state can be written as

$$|𝒢\rangle =|\xi \rangle_{A}⊗|\eta \rangle_{B}⊗|{𝒞}_{3}^{\prime }\rangle \;=\frac{1}{\sqrt{3}}(\alpha_{0}|0\rangle +\alpha_{1}|1\rangle {)}_{A}(\beta_{0}|0\rangle +\beta_{1}|1\rangle {)}_{B}\;\;\;⊗\sum_{j=0}^{2}|j\rangle_{1}\sum_{l=0}^{2}e^{2\pi ijl/3}a_{l}|ll\rangle_{23}\sum_{l=0}^{2}e^{2\pi ijl/3}b_{l}|ll\rangle_{45}\;=\frac{1}{2\sqrt{3}}\sum_{j=0}^{2}|j\rangle_{1}\{[\sum_{s=0}^{2}\sum_{t=0}^{1}|\Phi_{st}\rangle_{2A}\sum_{l=0}^{1}(-1{)}^{tl}\exp\{\frac{2\pi i}{3}j(s+l)mod3\}\alpha_{l}a_{(s+l)mod3}|(s+l)mod3\rangle_{3}]\;\;\;⊗[\sum_{m=0}^{2}\sum_{n=0}^{1}|\Phi_{mn}\rangle_{5B}\sum_{k=0}^{1}(-1{)}^{nk}\exp\{\frac{2\pi i}{3}j(m+k)mod3\}\beta_{k}b_{(m+k)mod3}|(m+k)mod3\rangle_{4}]\}.$$ (34)

To accomplish the QT task, Alice executes two-particle measurement on particle pair (2,*A*), with the basis $\left\{|\Phi_{st}\rangle \right\}$ as shown in Eq (11), and then informs Bob the measurement result $|\Phi_{st}\rangle_{2A}$ via the classical channel. At the same time, Bob also measures his particle pair (5,*B*) with the non-symmetric basis $\left\{|\Phi_{st}\rangle \right\}$, and tells Alice of his measurement result $|\Phi_{mn}\rangle_{5B}$ through a classical channel. Obviously, Alice and Bob randomly obtain a pair $\left(|\Phi_{st}\rangle_{2A},|\Phi_{mn}\rangle_{5B}\right)$ of measurement outcomes with the probability of $\frac{1}{4}[a_{s}^{2}|\alpha_{0}{|}^{2}\;+\;a_{(s+1)mod3}^{2}|\alpha_{1}{|}^{2}][b_{m}^{2}|\beta_{0}{|}^{2}\;+\;b_{(m+1)mod3}^{2}|\beta_{1}{|}^{2}]$, the state of qutrits 1, 3 and 4 collapses into

$$|{𝒢}^{\prime }\rangle =\frac{1}{\sqrt{3}}\sum_{j=0}^{2}|j\rangle_{1}\{[\sum_{l=0}^{1}(-1{)}^{tl}\exp\{\frac{2\pi i}{3}j(s+l)mod3\}\alpha_{l}a_{(s+l)mod3}|(s+l)mod3\rangle_{3}]\;\;\;⊗[\sum_{k=0}^{1}(-1{)}^{nk}\exp\{\frac{2\pi i}{3}j(m+k)mod3\}\beta_{k}b_{(m+k)mod3}|(m+k)mod3\rangle_{4}]\}.$$ (35)

If the supervisor Charlie agrees to cooperate, he needs to measure his qutrit 1 by using the computational basis {|0⟩,|1⟩,|2⟩}, and announce his measurement outcome $|h\rangle_{1}$ (h=0,1,2) to Alice and Bob. Charlie randomly gets measurement result $|h\rangle_{1}$ with the probability of 1/3, then the state of qutrits 3 and 4 will collapse into

$$|{𝒢}^{\prime \prime }\rangle =[\sum_{l=0}^{1}(-1{)}^{tl}\exp\{\frac{2\pi i}{3}h(s+l)mod3\}\alpha_{l}a_{(s+l)mod3}|(s+l)mod3\rangle_{3}]\;\;\;⊗[\sum_{k=0}^{1}(-1{)}^{nk}\exp\{\frac{2\pi i}{3}h(m+k)mod3\}\beta_{k}b_{(m+k)mod3}|(m+k)mod3\rangle_{4}].$$ (36)

After hearing the measurement messages, Alice carries out the unitary operation *U*_*mnh*_ as shown in Eq (30) on qutrit 4, at the same time, Bob also executes the unitary operation *U*_*sth*_ as shown in Eq (31) on qutrit 3. The result can be written as

$$|{𝒢}^{\prime \prime }\rangle =[\sum_{l=0}^{1}(-1{)}^{tl}\exp\{\frac{2\pi i}{3}h(s+l)mod3\}\alpha_{l}a_{(s+l)mod3}|(s+l)mod3\rangle_{3}]\;\;\;⊗[\sum_{k=0}^{1}(-1{)}^{nk}\exp\{\frac{2\pi i}{3}h(m+k)mod3\}\beta_{k}b_{(m+k)mod3}|(m+k)mod3\rangle_{4}].$$ (37)

To obtain the unknown state, Bob (Alice) introduces an auxiliary qubit $\hat{B}$ ($\hat{A}$) with the initial state $|0\rangle_{\hat{B}}$ ($|0\rangle_{\hat{A}}$) and executes one unitary transformation ${𝒰}_{sth}$ (${𝒰}_{mnh}$) under the basis $\{|00\rangle_{3\hat{B}},|01\rangle_{3\hat{B}},|10\rangle_{3\hat{B}}$, $|11\rangle_{3\hat{B}}\}$ ($\left\{|00\rangle_{4\hat{A}},|01\rangle_{4\hat{A}},|10\rangle_{4\hat{A}},|11\rangle_{4\hat{A}}\right\}$). Let $|\lambda_{sth}|=\min\{|a_{s}|,|a_{(s+1)mod3}|\}$, $|\lambda_{mnh}|=\min\{|b_{m}|$, $|b_{(m+1)mod3}|\}$, and take ${𝒰}_{sth}$ and ${𝒰}_{mnh}$ to be the 2×2 diagonal matrixes

$${𝒰}_{sth}=diag(W_{1},W_{2}),\;\;{𝒰}_{mnh}=diag(W_{3},W_{4}),$$ (38)

where for all s,m,h∈{0,1,2} and t,n∈{0,1},

$$W_{1}=\left(\begin{array}{cc} \lambda_{sth}/a_{s} & \sqrt{1-(\lambda_{sth}/a_{s}{)}^{2}}\\ -\sqrt{1-(\lambda_{sth}/a_{s}{)}^{2}} & \lambda_{sth}/a_{s} \end{array}\right),\;W_{2}=\left(\begin{array}{cc} \lambda_{sth}/a_{(s+1)mod3} & \sqrt{1-(\lambda_{sth}/a_{(s+1)mod3}{)}^{2}}\\ -\sqrt{1-(\lambda_{sth}/a_{(s+1)mod3}{)}^{2}} & \lambda_{sth}/a_{(s+1)mod3} \end{array}\right),\;W_{3}=\left(\begin{array}{cc} \lambda_{mnh}/b_{m} & \sqrt{1-(\lambda_{mnh}/b_{m}{)}^{2}}\\ -\sqrt{1-(\lambda_{mnh}/b_{m}{)}^{2}} & \lambda_{mnh}/b_{m} \end{array}\right),\;W_{4}=\left(\begin{array}{cc} \lambda_{mnh}/b_{(m+1)mod3} & \sqrt{1-(\lambda_{mnh}/b_{(m+1)mod3}{)}^{2}}\\ -\sqrt{1-(\lambda_{mnh}/b_{(m+1)mod3}{)}^{2}} & \lambda_{mnh}/b_{(m+1)mod3} \end{array}\right).$$ (39)

These two unitary transformation ${𝒰}_{sth}$ and ${𝒰}_{mnh}$ transform the state ${𝒰}_{mnh}$ into

$${𝒰}_{mnh}$$ (40)

Now, Bob (Alice) performs single-qubit measurement on the auxiliary qubit $\hat{B}$ ($\hat{A}$) in the *Z*-basis {|0⟩,|1⟩}. If Bob’s measurement result is $|0\rangle_{\hat{B}}$ with the probability of $\lambda_{sth}^{2}/[a_{s}^{2}|\alpha_{0}{|}^{2}+a_{(s+1)mod3}^{2}|\alpha_{1}{|}^{2}]$ and Alice’s result is $|0\rangle_{\hat{A}}$ with the probability of $\lambda_{mnh}^{2}/[b_{m}^{2}|\beta_{0}{|}^{2}+b_{(m+1)mod3}^{2}|\beta_{1}{|}^{2}]$, the state of qutrit 3 and 4 collapses into

$$(\alpha_{0}|0\rangle +\alpha_{1}|1\rangle {)}_{3}⊗(\beta_{0}|0\rangle +\beta_{1}|1\rangle {)}_{4},$$ (41)

which just happens to be the quantum states Alice and Bob want to exchange. Otherwise, it fails.

It can be seen from the above analysis that under the condition that Alice and Bob’s measurement results are $|\Phi_{st}\rangle_{2A}|0\rangle_{\hat{A}}$ and $|\Phi_{mn}\rangle_{5B}|0\rangle_{\hat{B}}$, respectively, the success probability of Alice and Bob exchanging their original unknown states is

$$\frac{1}{4}[a_{s}^{2}|\alpha_{0}{|}^{2}+a_{(s+1)mod3}^{2}|\alpha_{1}{|}^{2}][b_{m}^{2}|\beta_{0}{|}^{2}+b_{(m+1)mod3}^{2}|\beta_{1}{|}^{2}]\;\times \;\frac{1}{3}\;\;\times \;\frac{\lambda_{sth}^{2}}{a_{s}^{2}|\alpha_{0}{|}^{2}+a_{(s+1)mod3}^{2}|\alpha_{1}{|}^{2}}\;\times \;\frac{\lambda_{mnh}^{2}}{b_{m}^{2}|\beta_{0}{|}^{2}+b_{(m+1)mod3}^{2}|\beta_{1}{|}^{2}}\;=\frac{1}{12}\lambda_{sth}^{2}\lambda_{mnh}^{2}.$$ (42)

Therefore, the overall success probability of our scheme is $\frac{1}{4}\sum_{s,m=0}^{2}\sum_{t,n=0}^{1}\lambda_{sth}^{2}\lambda_{mnh}^{2}$.

Interestingly, when $a_{j}=b_{j}=1/\sqrt{3}$ (j=0,1,2), the states acting as a quantum channel, as shown in (33), becomes the maximally entangled states as shown in Eq (19), i.e. $|{𝒞}_{3}^{\prime }\rangle =|{𝒞}_{3}\rangle$. Meanwhile, $\lambda_{sth}=\lambda_{mnh}=1/\sqrt{3}$ and so the overall success probability of our scheme is $\frac{1}{4}\sum_{s,m=0}^{2}\sum_{t,n=0}^{1}\lambda_{sth}^{2}\lambda_{mnh}^{2}=\frac{1}{4}\;\times \;(3\;\times \;3\;\times \;2\;\times \;2)\times (\frac{1}{3}{)}^{2}\times (\frac{1}{3}{)}^{2}=1$. This shows that the scheme here at this time has become the previous standard controlled bidirectional teleportation. In other words, the controlled bidirectional teleportation scheme using a non-maximally entangled 5-qutrit state as quantum channel is the generalization of the scheme in the first part of this section.

**Remark** (i) In this section we obtain two schemes, one deterministic and the other probabilistic. Compared with the quantum states shown in Eqs (25), (26), (32) and (41), the fidelities of our schemes are 1.

(ii) Since we employ non-symmetric basis measurement and use a 3-dimensional entangled 5-qutrit state as quantum channel, the controlled Bidirectional teleportation is realized between the two parts of different dimensions under the control of the supervisor, which makes our schemes different from the known ones for CBT of two unknown single-qubit states.

(iii) We give the general mathematical formulations for a series of operations on both the sender, the receiver and the supervisor, which accurately and concisely depicts the intrinsic connections among a series of operations in a communication scheme, greatly simplifies the cumbersome description that the schemes should originally have, and are easy to generalize the schemes.

## 4 CBT of unknown single-qubit states via a *d*-dimensional entangled state

### 4.1 CBT based on the *d*-dimensional maximally entangled state

In this subsection, we generalized the above schemes to a *d*-dimensional entangled states as quantum channels. Suppose the quantum states that Alice and Bob want to exchange are still unknown 2-dimensional single-qubit states $|\xi \rangle_{A}$ and $|\eta \rangle_{B}$ as shown in Eqs (25) and (26). Alice, Bob and Charlie priori share a *d*-dimensional maximally entangled 5-qudit state $|{𝒞}_{d}\rangle$ as shown in Eq (24) in such way that qudit 1 belongs to Charlie, qudits 2 and 4 belong to Alice, and qudits 3 and 5 belong to Bob, respectively. The state of the entire initial system can be written

$$|𝒬\rangle =|\xi \rangle_{A}⊗|\eta \rangle_{b}⊗|{𝒞}_{d}\rangle \;=\frac{1}{d\sqrt{d}}(\alpha_{0}|0\rangle +\alpha_{1}|1\rangle {)}_{A}⊗(\beta_{0}|0\rangle +\beta_{1}|1\rangle {)}_{B}\;\;\;⊗\sum_{j=0}^{d-1}|j\rangle_{1}\sum_{l=0}^{d-1}e^{2\pi ijl/d}|ll\rangle_{23}\sum_{l=0}^{2}e^{2\pi ijl/d}|ll\rangle_{45}\;=\frac{1}{2d\sqrt{d}}\sum_{j=0}^{d-1}|j\rangle_{1}\{[\sum_{s=0}^{d-1}\sum_{t=0}^{1}|\Psi_{st}\rangle_{2A}\sum_{l=0}^{1}(-1{)}^{tl}\exp\{\frac{2\pi i}{d}j(s+l)modd\}\alpha_{l}|(s+l)modd\rangle_{3}]\;\;\;⊗[\sum_{m=0}^{d-1}\sum_{n=0}^{1}|\Psi_{mn}\rangle_{5B}\sum_{k=0}^{1}(-1{)}^{nk}\exp\{\frac{2\pi i}{d}j(m+k)modd\}\beta_{k}|(m+k)modd\rangle_{4}]\}.$$ (43)

To realize the controlled Bidirectional teleportation, Alice uses the non-symmetric basis $\left\{|\Psi_{kl}\rangle \right\}$ as shown in Eq (15) to measure particle pair (2,*A*), and then tells Bob about her measurement result $|\Psi_{st}\rangle_{2A}$ through the classical channel. Meanwhile, Bob measures his particle pair (5,*B*) with the non-symmetric basis $\left\{|\Psi_{kl}\rangle \right\}$, and informs Alice of his measurement result $|\Psi_{mn}\rangle_{5B}$ via the classical channel. Clearly, from Eq (43), the probability of obtaining the joint measurement outcome $|\Psi_{st}\rangle_{2A}|\Psi_{mn}\rangle_{5B}$ of Alice and Bob is 1/4*d*^2^, and the state of qudits 1, 3 and 4 will collapse into

$$|{𝒬}^{\prime }\rangle =\frac{1}{\sqrt{d}}\sum_{j=0}^{d-1}|j\rangle_{1}\{[\sum_{l=0}^{1}(-1{)}^{tl}\exp\{\frac{2\pi i}{d}j(s+l)modd\}\alpha_{l}|(s+l)modd\rangle_{3}]\;\;\;⊗[\sum_{k=0}^{1}(-1{)}^{nk}\exp\{\frac{2\pi i}{d}j(m+k)modd\}\beta_{k}|(m+k)modd\rangle_{4}]\}.$$ (44)

If Charlie agrees to cooperate with Alice and Bob, he needs to carry out a single-qudit measurement on his qudit 1 with the computational basis {|0⟩,|1⟩,⋯,|d−1⟩}, and then publicly announces his measurement result $|h\rangle_{1}$ (h=0,1,⋯,d−1) to Alice and Bob through classical communication. Form Eq (44), Charlie gets result $|h\rangle_{1}$ with the probability of 1/*d*, and the state of qudits 3 and 4 will collapse into

$$|{𝒬}^{\prime \prime }\rangle =[\sum_{l=0}^{1}(-1{)}^{tl}\exp\{\frac{2\pi i}{d}h(s+l)modd\}\alpha_{l}|(s+l)modd\rangle_{3}]\;\;\;⊗[\sum_{k=0}^{1}(-1{)}^{nk}\exp\{\frac{2\pi i}{d}h(m+k)modd\}\beta_{k}|(m+k)modd\rangle_{4}].$$ (45)

In accordance with Alice and Charlie’s public announcement of thier measurement results, Bob may execute a unitary operation

$$U_{sth}^{\left(d\right)}=\exp\{\frac{-2\pi i}{d}hs\}|0\rangle \langle s|+(-1{)}^{t}\exp\{\frac{-2\pi i}{d}h(s+1)modd\}|1\rangle \langle (s+1)modd|$$ (46)

on qudit 3. At the same time, Alice performs a unitary operation

$$U_{mnh}^{\left(d\right)}=\exp\{\frac{-2\pi i}{d}hm\}|0\rangle \langle m|+(-1{)}^{n}\exp\{\frac{-2\pi i}{d}h(m+1)modd\}|1\rangle \langle (m+1)modd|$$ (47)

on qudit 4 after hearing Bob and Charlie’s measurement information. These two unitary operations evolve the state $|{𝒬}^{\prime \prime }\rangle$ into

$$(U_{sth}^{\left(d\right)}\;⊗\;U_{mnh}^{\left(d\right)})|{𝒬}^{\prime \prime }\rangle =(\alpha_{0}|0\rangle +\alpha_{1}|1\rangle {)}_{3}⊗(\beta_{0}|0\rangle +\beta_{1}|1\rangle {)}_{4}.$$ (48)

Eq (48) indicates that Alice and Bob has successfully exchanged the original unknown single-qubit states with the probability of 1/4*d*^3^ under the control of the supervisor Charlie.

Noting that s,m,h∈{0,1,⋯,d−1} and t,n∈{0,1}, the overall success probability of our scheme here is

$$\frac{1}{4d^{3}}\;\times \;(d\;\times \;d\;\times \;d\;\times \;2\;\times \;2)=1,$$ (49)

i.e., this scheme is a deterministic scheme.

### 4.2 CBT based on the *d*-dimensional non-maximally entangled state

Now, we also consider the possibility of realizing the controlled bidirectional teleportation based on the *d*-dimensional non-maximally entangled 5-qudit state as the quantum channel. Suppose that Alice and Bob pre-share a *d*-dimensional non-maximally entangled state given by

$$|{𝒞}_{d}^{\prime }\rangle =\frac{1}{\sqrt{d}}\sum_{j=0}^{d-1}|j\rangle_{1}\sum_{l=0}^{d-1}e^{2\pi ijl/d}a_{l}|ll\rangle_{23}\sum_{l=0}^{2}e^{2\pi ijl/d}b_{l}|ll\rangle_{45}.$$ (50)

where real numbers *a*_*j*_ and *b*_*j*_ (j=0,1,⋯,d−1) satisfy $\sum_{j=0}^{d-1}a_{j}^{2}=1$ and $\sum_{j=0}^{d-1}b_{j}^{2}=1$. Qudit 1 belongs to Charlie, qudits 2 and 4 belong to Alice, and qudits 3 and 5 belong to Bob, respectively. Alice wishes to teleport the state $|\xi \rangle_{A}$ as shown in Eq (25) to distant Bob, at the same time, Bob wants to teleport the state $|\eta \rangle_{B}$ as shown in Eq (26) to Alice under the control of Charlie. The state of whole initial system may be expressed as

$$|Q\rangle =|\xi \rangle_{A}⊗|\eta \rangle_{B}⊗|{𝒞}_{d}^{\prime }\rangle \;=(\alpha_{0}|0\rangle +\alpha_{1}|1\rangle {)}_{A}⊗(\beta_{0}|0\rangle +\beta_{1}|1\rangle {)}_{B}\;\;\;⊗\frac{1}{\sqrt{d}}\sum_{j=0}^{d-1}|j\rangle_{1}\sum_{l=0}^{d-1}e^{2\pi ijl/d}a_{l}|ll\rangle_{23}\sum_{l=0}^{2}e^{2\pi ijl/d}b_{l}|ll\rangle_{45}\;=\frac{1}{2\sqrt{d}}\sum_{j=0}^{d-1}|j\rangle_{1}\{[\sum_{s=0}^{d-1}\sum_{t=0}^{1}|\Psi_{st}\rangle_{2A}\sum_{l=0}^{1}(-1{)}^{tl}\exp\{\frac{2\pi i}{d}j(s+l)modd\}\alpha_{l}a_{(s+l)modd}|(s+l)modd\rangle_{3}]\;\;\;⊗[\sum_{m=0}^{d-1}\sum_{n=0}^{1}|\Psi_{mn}\rangle_{5B}\sum_{k=0}^{1}(-1{)}^{nk}\exp\{\frac{2\pi i}{d}j(m+k)modd\}\beta_{k}b_{(m+k)modd}|(m+k)modd\rangle_{4}]\}.$$ (51)

To finish the quantum task, Alice needs to measure her particle pair (2,*A*) with the non-symmetric basis $\left\{|\Psi_{kl}\rangle \right\}$ as shown in Eq (15), and then publishes her measurement outcome $|\Psi_{st}\rangle_{2A}$ to Bob. Meanwhile, Bob also performs a von Neumann measurement on the particle pair (5,*A*) with the non-symmetric basis $\left\{|\Psi_{kl}\rangle \right\}$, and tells Alice of his outcome $|\Psi_{mn}\rangle_{5B}$.

Obviously, Alice and Bob can randomly obtain a set $\left(|\Psi_{st}\rangle_{2A},|\Psi_{mn}\rangle_{5B}\right)$ of measurement outcomes with probability $\left(1/4)[\sum_{l=0}^{1}|\alpha_{l}{|}^{2}a_{(s+l)modd}^{2}\sum_{k=0}^{1}|\beta_{k}{|}^{2}b_{(m+k)modd}^{2}\right]$, the state of qudits 3 and 4 will collapse into

$$|Q^{\prime }\rangle =\frac{1}{\sqrt{d}}\sum_{j=0}^{d-1}|j\rangle_{1}\{[\sum_{l=0}^{1}(-1{)}^{tl}\exp\{\frac{2\pi i}{d}j(s+l)modd\}\alpha_{l}a_{(s+l)modd}|(s+l)modd\rangle_{3}]\;\;\;⊗[\sum_{k=0}^{1}(-1{)}^{nk}\exp\{\frac{2\pi i}{d}j(m+k)modd\}\beta_{k}b_{(m+k)modd}|(m+k)modd\rangle_{4}]\}.$$ (52)

If Charlie is willing to cooperate with Alice and Bob, he should perform a von Neumann measurement on his qudit 1 with respect to the computational basis {|0⟩,|1⟩,⋯,|d−1⟩} and publishes his measurement outcome $|h\rangle_{1}$ (h=0,1,⋯,d−1) to Alice and Bob via classical communication. Then, the state of qudits 3 and 4 will collapse into

$$|Q^{\prime \prime }\rangle =[\sum_{l=0}^{1}(-1{)}^{tl}\exp\{\frac{2\pi i}{d}h(s+l)modd\}\alpha_{l}a_{(s+l)modd}|(s+l)modd\rangle_{3}]\;\;\;⊗[\sum_{k=0}^{1}(-1{)}^{nk}\exp\{\frac{2\pi i}{d}h(m+k)modd\}\beta_{k}b_{(m+k)modd}|(m+k)modd\rangle_{4}]$$ (53)

with probability 1/*d*.

According to the measurement information, Bob and Alice perform the unitary operations $U_{sth}^{\left(d\right)}$ and $U_{mnh}^{\left(d\right)}$ as shown in Eqs (46) and (47) on qudits 3 and 4, respectively, which transform the state $|Q^{\prime \prime }\rangle$ into

$$|Q^{\prime \prime }\rangle$$ (54)

Then, Bob (Alice) introduces an auxiliary qubit $B^{\prime }$ ($A^{\prime }$) with the initial state $|0\rangle_{B^{\prime }}$ ($|0\rangle_{A^{\prime }}$) and executes one unitary transformation ${𝒰}_{sth}^{\prime }$ (${𝒰}_{mnh}^{\prime }$) under the basis $\{|00\rangle_{3B^{\prime }},|01\rangle_{3B^{\prime }},|10\rangle_{3B^{\prime }}$, $|11\rangle_{3B^{\prime }}\}$ ($\{|00\rangle_{4A^{\prime }}$, $|01\rangle_{4A^{\prime }},|10\rangle_{4A^{\prime }},|11\rangle_{4A^{\prime }}\}$). Let $|\lambda_{sth}^{\prime }|=\min\{|a_{s}|,|a_{(s+1)modd}|\}$, $|\lambda_{mnh}^{\prime }|=\min\{|b_{m}|$, $|b_{(m+1)modd}|\}$, and take ${𝒰}_{sth}^{\prime }$ and ${𝒰}_{mnh}^{\prime }$ to be the 2×2 diagonal matrixes

$${𝒰}_{sth}^{\prime }=diag(W_{1}^{\prime },W_{2}^{\prime }),\;\;{𝒰}_{mnh}^{\prime }=diag(W_{3}^{\prime },W_{4}^{\prime }),$$ (55)

where for all s,m,h∈{0,1,⋯,d−1} and t,n∈{0,1},

$$W_{1}^{\prime }=\left(\begin{array}{cc} \lambda_{sth}^{\prime }/a_{s} & \sqrt{1-(\lambda_{sth}^{\prime }/a_{s}{)}^{2}}\\ -\sqrt{1-(\lambda_{sth}^{\prime }/a_{s}{)}^{2}} & \lambda_{sth}^{\prime }/a_{s} \end{array}\right),\;W_{2}^{\prime }=\left(\begin{array}{cc} \lambda_{sth}^{\prime }/a_{(s+1)modd} & \sqrt{1-(\lambda_{sth}^{\prime }/a_{(s+1)modd}{)}^{2}}\\ -\sqrt{1-(\lambda_{sth}^{\prime }/a_{(s+1)modd}{)}^{2}} & \lambda_{sth}^{\prime }/a_{(s+1)modd} \end{array}\right),\;W_{3}^{\prime }=\left(\begin{array}{cc} \lambda_{mnh}^{\prime }/b_{m} & \sqrt{1-(\lambda_{mnh}^{\prime }/b_{m}{)}^{2}}\\ -\sqrt{1-(\lambda_{mnh}^{\prime }/b_{m}{)}^{2}} & \lambda_{mnh}^{\prime }/b_{m} \end{array}\right),\;W_{4}^{\prime }=\left(\begin{array}{cc} \lambda_{mnh}^{\prime }/b_{(m+1)modd} & \sqrt{1-(\lambda_{mnh}^{\prime }/b_{(m+1)modd}{)}^{2}}\\ -\sqrt{1-(\lambda_{mnh}^{\prime }/b_{(m+1)modd}{)}^{2}} & \lambda_{mnh}^{\prime }/b_{(m+1)modd} \end{array}\right).$$ (56)

These two unitary transformation ${𝒰}_{sth}^{\prime }$ and ${𝒰}_{mnh}^{\prime }$ transform the state ${𝒰}_{mnh}^{\prime }$ into

$${𝒰}_{mnh}^{\prime }$$ (57)

Subsequently, Alice and Bob respectively measure the auxiliary qubits $A^{\prime }$ and $B^{\prime }$ with the computational basis {|0⟩,|1⟩}. If Alice and Bob’s measurement results are $|0\rangle_{A^{\prime }}$ and $|0\rangle_{B^{\prime }}$ with the probability $\lambda_{mnh}^{\prime 2}/[b_{m}^{2}|\beta_{0}{|}^{2}\;+\;b_{(m+1)modd}^{2}|\beta_{1}{|}^{2}]$ and $\lambda_{sth}^{\prime 2}/[a_{s}^{2}|\alpha_{0}{|}^{2}\;+\;a_{(s+1)modd}^{2}|\alpha_{1}{|}^{2}]$, respectively, the state of qudits 3 and 4 will be collapsed into $(\alpha_{0}|0\rangle \;+\;\alpha_{1}|1\rangle {)}_{3}\;⊗\;(\beta_{0}|0\rangle \;+\;\beta_{1}|1\rangle {)}_{4}$. Otherwise, the teleportation fails. That is, on the conditions that Alice and Bob’s outcomes respectively are $|\Psi_{st}\rangle_{2A}|0\rangle_{A^{\prime }}$ and $|\Psi_{mn}\rangle_{5B}|0\rangle_{B^{\prime }}$, and Charlie’s outcome is $|h\rangle_{1}$, Alice and Bob can exchange their original unknown sing-qubit states with the probability

$$\frac{1}{4}[\sum_{l=0}^{1}|\alpha_{l}{|}^{2}a_{(s+l)modd}^{2}\sum_{k=0}^{1}|\beta_{k}{|}^{2}b_{(m+k)modd}^{2}]\times \frac{1}{d}\;\;\times \;\frac{\lambda_{mnh}^{\prime 2}}{b_{m}^{2}|\beta_{0}{|}^{2}+b_{(m+1)modd}^{2}|\beta_{1}{|}^{2}}\times \frac{\lambda_{sth}^{\prime 2}}{a_{s}^{2}|\alpha_{0}{|}^{2}+a_{(s+1)modd}^{2}|\alpha_{1}{|}^{2}}\;=\frac{1}{4d}\lambda_{sth}^{\prime 2}\lambda_{mnh}^{\prime 2}.$$ (58)

Since s,m,h∈{0,1,⋯,d−1} and t,n∈{0,1}, the overall success probability of our scheme here is $\frac{1}{4d}\sum_{s,m,h=0}^{d-1}\sum_{t,n=0}^{1}\lambda_{sth}^{\prime 2}\lambda_{mnh}^{\prime 2}$.

It is worth noting that when $a_{j}=b_{j}=1/\sqrt{d}$ (j=0,1,⋯,d−1), $|{𝒞}_{d}^{\prime }\rangle =|{𝒞}_{d}\rangle$, and $\lambda_{sth}^{\prime }=\lambda_{mnh}^{\prime }=1/\sqrt{d}$ for any s,m,h∈{0,1,⋯,d−1} and t,n∈{0,1}, and so $\frac{1}{4d}\sum_{s,m,h=0}^{d-1}\sum_{t,n=0}^{1}\lambda_{sth}^{\prime 2}\lambda_{mnh}^{\prime 2}=\frac{1}{4d}\;\times \;(\frac{1}{d}\;\times \;\frac{1}{d})\;\times \;(d\;\times \;d\;\times \;d\;\times \;2\;\times \;2)=1$. At this time, the scheme here is exactly the same as scheme in the previous part of this section. In this sense, the scheme in the second half of this section is the generalization of that in the first half.

## 5 CBT of unknown *f*-dimensional single-particle states via a *d*-dimensional entangled state

### 5.1 CBT based on *f*-dimensional single-particle states by using a *d*-dimensional maximally entangled state

In this subsection, we consider to exchange two *f*-dimensional single-particle states by using a *d*-dimensional maximally or non-maximally entangled state under the control of a supervisor, which generalize the schemes in the section 4.

Assume that Alice has an unknown *f*-dimensional single-particle state $|\zeta \rangle_{A}$ to teleport to Bob, which is given by

$$|\zeta \rangle_{A}=\sum_{j=0}^{f-1}\alpha_{j}|j\rangle_{A},$$ (59)

where complex numbers $\alpha_{0},\alpha_{1},\cdots ,\alpha_{f-1}$ satisfy the normalization condition $\sum_{j=0}^{f-1}|\alpha_{j}{|}^{2}=1$. At the same time, Bob has also an unknown *f*-dimensioal single-particle state $|\varsigma \rangle_{B}$, which is written as

$$|\varsigma \rangle_{B}=\sum_{j=0}^{f-1}\beta_{j}|j\rangle_{B},$$ (60)

where $\beta_{0},\beta_{1},\cdots ,\beta_{f-1}$ are complex numbers with $\sum_{j=0}^{f-1}|\beta_{j}{|}^{2}=1$, he wants to teleport this state to Alice under the control of a supervisor Charlie. Beforehand, Alice, Bob and Charlie share a *d*-dimensional maximally entangled 5-qudit state $|{𝒞}_{d}\rangle$ as shown in Eq (24), where qudit 1 belongs to Charlie, qudits 2 and 4 belong to Alice, and qudits 3 and 5 belong to Bob, respectively. The state of whole initial system can be written as

$$|T\rangle =|\zeta \rangle_{A}⊗|\varsigma \rangle_{B}⊗|{𝒞}_{d}\rangle \;=\frac{1}{d\sqrt{d}}\sum_{j=0}^{f-1}\alpha_{j}|j\rangle_{A}⊗\sum_{j=0}^{f-1}\beta_{j}|j\rangle_{B}\;\;\;⊗\sum_{j=0}^{d-1}|j\rangle_{1}\sum_{l=0}^{d-1}e^{2\pi ijl/d}|ll\rangle_{23}\sum_{l=0}^{2}e^{2\pi ijl/d}|ll\rangle_{45}\;=\frac{1}{fd\sqrt{d}}\sum_{j=0}^{d-1}|j\rangle_{1}\{[\sum_{s=0}^{d-1}\sum_{t=0}^{f-1}|\Upsilon_{st}\rangle_{2A}\sum_{l=0}^{f-1}\exp\{2\pi i(\frac{j(l+s)modd}{d}-\frac{tl}{f})\}\alpha_{l}|(l+s)modd\rangle_{3}]\;\;\;⊗[\sum_{m=0}^{d-1}\sum_{n=0}^{f-1}|\Upsilon_{mn}\rangle_{5B}\sum_{k=0}^{f-1}\exp\{2\pi i(\frac{j(k+m)modd}{d}-\frac{nk}{f})\}\beta_{k}|(k+m)modd\rangle_{4}]\}.$$ (61)

To fulfil this quantum task, Alice and Bob use the non-symmetric basis $\left\{|\Upsilon_{st}\rangle \right\}$ as shown in Eq (18) to measure their particle pairs (2,*A*) and (5,*B*), respectively, and inform each other of their respective measurement information through classical communication. If Alice and Bob’s measurement results are $|\Upsilon_{st}\rangle_{2A}$ and $|\Upsilon_{mn}\rangle_{5B}$, respectively, the state of qudits 1, 3 and 4 will collapse into

$$|T^{\prime }\rangle =\frac{1}{\sqrt{d}}\sum_{j=0}^{d-1}|j\rangle_{1}\{[\sum_{l=0}^{f-1}\exp\{2\pi i(\frac{j(l+s)modd}{d}-\frac{tl}{f})\}\alpha_{l}|(l+s)modd\rangle_{3}]\;\;\;⊗[\sum_{k=0}^{f-1}\exp\{2\pi i(\frac{j(k+m)modd}{d}-\frac{nk}{f})\}\beta_{k}|(k+m)modd\rangle_{4}]\}.$$ (62)

If Charlie is willing to cooperate with Alice and Bob, he should measure his qudit 1 in the computational basis {|0⟩,|1⟩,⋯,|d−1⟩} and announce his measurement outcome $|h\rangle_{1}$ to Alice and Bob though classical channels. Then the state of qudits 3 and 4 collapses into

$$|T^{\prime \prime }\rangle =[\sum_{l=0}^{f-1}\exp\{2\pi i(\frac{h(l+s)modd}{d}-\frac{tl}{f})\}\alpha_{l}|(l+s)modd\rangle_{3}]\;\;\;⊗[\sum_{k=0}^{f-1}\exp\{2\pi i(\frac{h(k+m)modd}{d}-\frac{nk}{f})\}\beta_{k}|(k+m)modd\rangle_{4}].$$ (63)

Based on the measurement information, Alice applies the local operation

$${\tilde{U}}_{mnh}=\sum_{k=0}^{f-1}\exp\{2\pi i(\frac{nk}{f}-\frac{h(k+m)modd}{d})\}|k\rangle \langle (k+m)modd|$$ (64)

to qudit 4, Bob also applies the local operation

$${\tilde{U}}_{sth}=\sum_{l=0}^{f-1}\exp\{2\pi i(\frac{tl}{f}-\frac{h(l+s)modd}{d})\}|l\rangle \langle (l+s)modd|$$ (65)

to qudit 3, i.e.,

$$({\tilde{U}}_{mnh}⊗{\tilde{U}}_{sth})|T^{\prime \prime }\rangle =[\sum_{l=0}^{f-1}\alpha_{l}|l\rangle_{3}]⊗[\sum_{k=0}^{f-1}\beta_{k}|k\rangle_{4}].$$ (66)

From Eqs (61) and (66), one can see that under the control of the supervisor Charlie, Alice and Bob successfully exchange their quantum states with the probability of $(1/fd\sqrt{d}{)}^{2}=1/f^{2}d^{3}$. Since s,m,h∈{0,1,⋯,d−1} and t,n∈{0,1,⋯,f−1}, therefore the overall success probability of our scheme is


$$\frac{1}{f^{2}d^{3}}\;\times \;(d\;\times \;d\;\times \;d\;\times \;f\;\times \;f)=1.$$

### 5.2 CBT based on *f*-dimensional single-particle states by using a *d*-dimensional non-maximally entangled state

Now, we consider the CBT of two *f*-dimensional single-particle states by using a *d*-dimensional non-maximally entangled 5-qudit state as quantum channel. Supposing the states that Alice and Bob want to exchange are still $|\zeta \rangle_{A}$ and $|\varsigma \rangle_{B}$ as shown in Eqs (59) and (60). Alice, Bob and Charlie pre-share a *d*-dimensional non-maximally entangled 5-qudit state $|{𝒞}_{d}^{\prime }\rangle$ as shown in Eq (50) in such way that qudit 1 belongs to Charlie, qudits 2 and 4 belong to Alice, and qudits 3 and 5 belong to Bob. Thus the state of whole initial system can be expressed as

$$|𝒯\rangle =|\zeta \rangle_{A}⊗|\varsigma \rangle_{B}⊗|{𝒞}_{d}^{\prime }\rangle \;=\frac{1}{\sqrt{d}}\sum_{j=0}^{f-1}\alpha_{j}|j\rangle_{A}⊗\sum_{j=0}^{f-1}\beta_{j}|j\rangle_{B}⊗\sum_{j=0}^{d-1}|j\rangle_{1}\sum_{l=0}^{d-1}e^{2\pi ijl/d}a_{l}|ll\rangle_{23}\sum_{l=0}^{2}e^{2\pi ijl/d}b_{l}|ll\rangle_{45}\;=\frac{1}{f\sqrt{d}}\sum_{j=0}^{d-1}|j\rangle_{1}\{[\sum_{s=0}^{d-1}\sum_{t=0}^{f-1}|\Upsilon_{st}\rangle_{2A}\sum_{l=0}^{f-1}\exp\{2\pi i(\frac{j(l+s)modd}{d}-\frac{tl}{f})\}\alpha_{l}a_{(l+s)modd}|(l+s)modd\rangle_{3}]\;\;\;⊗[\sum_{m=0}^{d-1}\sum_{n=0}^{f-1}|\Upsilon_{mn}\rangle_{5B}\sum_{k=0}^{f-1}\exp\{2\pi i(\frac{j(k+m)modd}{d}-\frac{nk}{f})\}\beta_{k}b_{(k+m)modd}|(k+m)modd\rangle_{4}]\}.$$ (67)

To accomplish the CBT task, Alice and Bob measure their particle pairs (2,*A*) and (5,*B*), respectively, with respect to the non-symmetric basis $\left\{|\Upsilon_{st}\rangle \right\}$ as shown in Eq (18), and exchange their measurement outcomes via classical communication. If Alice and Bob’s measurement outcomes are $|\Upsilon_{st}\rangle_{2A}$ and $|\Upsilon_{mn}\rangle_{5B}$, respectively, the state of qudits 1, 3 and 4 collapses into

$$|{𝒯}^{\prime }\rangle =\frac{1}{\sqrt{d}}\sum_{j=0}^{d-1}|j\rangle_{1}\{[\sum_{l=0}^{f-1}\exp\{2\pi i(\frac{j(l+s)modd}{d}-\frac{tl}{f})\}\alpha_{l}a_{(l+s)modd}|(l+s)modd\rangle_{3}]\;\;\;⊗[\sum_{k=0}^{f-1}\exp\{2\pi i(\frac{j(k+m)modd}{d}-\frac{nk}{f})\}\beta_{k}b_{(k+m)modd}|(k+m)modd\rangle_{4}]\}.$$ (68)

When Charlie agrees to work with Alice and Bob, he needs to measure his qudit 1 in the *Z*-basis {|h⟩:h=0,1,⋯,d−1} and communicate his measurement outcome $|h\rangle_{1}$ to Alice and Bob via classical communication. Then the state of qudits 3 and 4 collapses into

$$|{𝒯}^{\prime \prime }\rangle =[\sum_{l=0}^{f-1}\exp\{2\pi i(\frac{h(l+s)modd}{d}-\frac{tl}{f})\}\alpha_{l}a_{(l+s)modd}|(l+s)modd\rangle_{3}]\;\;\;⊗[\sum_{k=0}^{f-1}\exp\{2\pi i(\frac{h(k+m)modd}{d}-\frac{nk}{f})\}\beta_{k}b_{(k+m)modd}|(k+m)modd\rangle_{4}].$$ (69)

From Eq (67), the probability that Alice, Bob and Charlie get $|\Upsilon_{st}\rangle_{2A}$, $|\Upsilon_{mn}\rangle_{5B}$ and $|h\rangle_{1}$ respectively is

$$\frac{1}{f^{2}d}[\sum_{l=0}^{f-1}|\alpha_{l}{|}^{2}a_{(l+s)modd}^{2}][\sum_{k=0}^{f-1}|\beta_{k}{|}^{2}b_{(k+m)modd}^{2}].$$ (70)

According to measurement information, Bob and Alice apply the unitary operations ${\tilde{U}}_{sth}$ and ${\tilde{U}}_{mnh}$ as shown in Eqs (64) and (65) to qudits 3 and 4, respectively, we have

$${\tilde{U}}_{mnh}$$ (71)

Then, Bob (Alice) introduces an auxiliary qubit $B^{\prime \prime }$ ($A^{\prime \prime }$) with the initial state $|0\rangle_{B^{\prime \prime }}$ ($|0\rangle_{A^{\prime \prime }}$) and executes one unitary transformation ${𝒰}_{sth}^{\prime \prime }$ (${𝒰}_{mnh}^{\prime \prime }$) under the basis $\{|00\rangle_{3B^{\prime \prime }},|01\rangle_{3B^{\prime \prime }},|10\rangle_{3B^{\prime \prime }}$, $|11\rangle_{3B^{\prime \prime }}$, ⋯, $|d-1,0\rangle_{3B^{\prime \prime }}$, $|d-1,1\rangle_{3B^{\prime \prime }}\}$ ($\{|00\rangle_{4A^{\prime \prime }}$, $|01\rangle_{4A^{\prime \prime }}$, $|10\rangle_{4A^{\prime \prime }}$, $|11\rangle_{4A^{\prime \prime }}$, ⋯, $|d-1,0\rangle_{4A^{\prime \prime }}$, $|d-1,1\rangle_{4A^{\prime \prime }}\}$). Let $|\lambda_{sth}^{\prime \prime }|=\min\{|a_{s}|$, $|a_{(s+1)modd}|$, ⋯, $|a_{(s+f-1)modd}|\}$, $|\lambda_{mnh}^{\prime \prime }|=\min\{|b_{m}|$, $|b_{(m+1)modd}|$, ⋯, $|b_{(m+f-1)modd}|\}$, and take ${𝒰}_{sth}^{\prime \prime }$ and ${𝒰}_{mnh}^{\prime \prime }$ to be the f×f diagonal matrixes

$${𝒰}_{sth}^{\prime \prime }=diag({\hat{W}}_{0},{\hat{W}}_{1},\cdots ,{\hat{W}}_{f-1}),\;\;{𝒰}_{mnh}^{\prime \prime }=diag({\tilde{W}}_{0},{\tilde{W}}_{1},\cdots ,{\tilde{W}}_{f-1}),$$ (72)

where for any j∈{0,1,⋯,f−1},

$${\hat{W}}_{j}=\left(\begin{array}{cc} \lambda_{sth}^{\prime \prime }/a_{(s+j)modd} & \sqrt{1-(\lambda_{sth}^{\prime \prime }/a_{(s+j)modd}{)}^{2}}\\ -\sqrt{1-(\lambda_{sth}^{\prime \prime }/a_{(s+j)modd}{)}^{2}} & \lambda_{sth}^{\prime \prime }/a_{(s+j)modd} \end{array}\right),\;{\tilde{W}}_{j}=\left(\begin{array}{cc} \lambda_{mnh}^{\prime \prime }/b_{(m+j)modd} & \sqrt{1-(\lambda_{mnh}^{\prime \prime }/b_{(m+j)modd}{)}^{2}}\\ -\sqrt{1-(\lambda_{mnh}^{\prime \prime }/b_{(m+j)modd}{)}^{2}} & \lambda_{mnh}^{\prime \prime }/b_{(m+j)modd} \end{array}\right).$$ (73)

These two unitary transformation ${𝒰}_{sth}^{\prime \prime }$ and ${𝒰}_{mnh}^{\prime \prime }$ transform the state ${𝒰}_{mnh}^{\prime \prime }$ into

$${𝒰}_{mnh}^{\prime \prime }$$ (74)

Subsequently, Alice and Bob respectively measure the auxiliary qubits $A^{\prime \prime }$ and $B^{\prime \prime }$ with the computational basis {|0⟩,|1⟩}. If Alice and Bob’s measurement results are $|0\rangle_{A^{\prime \prime }}$ and $|0\rangle_{B^{\prime \prime }}$ with the probability $|0\rangle_{B^{\prime \prime }}$ and $|0\rangle_{B^{\prime \prime }}$, respectively, the state of qudits 3 and 4 will be collapsed into $\left(\sum_{l=0}^{f-1}\alpha_{l}|l\rangle_{3})\;⊗\;(\sum_{k=0}^{f-1}\beta_{k}|k\rangle_{4}\right)$. Otherwise, the teleportation fails. That is, on the conditions that Alice and Bob’s outcomes respectively are $|\Upsilon_{st}\rangle_{2A}|0\rangle_{A^{\prime \prime }}$ and $|\Upsilon_{mn}\rangle_{5B}|0\rangle_{B^{\prime \prime }}$, and Charlie’s outcome is $|h\rangle_{1}$, Alice and Bob can exchange their original unknown *f*-dimensional sing-qubit states with the probability

$$|h\rangle_{1}$$ (75)

Since s,m,h∈{0,1,⋯,d−1} and t,n∈{0,1}, the overall success probability of our scheme here is $[\sum_{s,m,h=0}^{d-1}\sum_{t,n=0}^{1}\lambda_{sth}^{\prime 2}\lambda_{mnh}^{\prime 2}]/f^{2}d$. It is worth noting that when $a_{j}=b_{j}=1/\sqrt{d}$ (j=0,1,⋯,d−1), $|{𝒞}_{d}^{\prime }\rangle =|{𝒞}_{d}\rangle$, and $\lambda_{sth}^{\prime \prime }=\lambda_{mnh}^{\prime \prime }=1/\sqrt{d}$ for any s,m,h∈{0,1,⋯,d−1} and t,n∈{0,1,⋯.f−1}, and so


t,n∈{0,1,⋯.f−1}

At this time, the scheme here is exactly the same as scheme in the previous part of this section. In this sense, the scheme in the second half of this section is the generalization of that in the first half.

## 6 Discussion and conclusion

We present two teleportation protocols for arbitrary unknown two-qubit states utilizing high-dimensional entangled two-qudit channels. Detailed analysis is provided for the special case employing three-dimensional two-qutrit entangled channels. Notably, both protocols exhibit enhanced security. To clarify this feature, we incorporate a security verification procedure: The sender Alice prepares a verification sequence comprising randomly *Z*-basis |0⟩,|1⟩,|+⟩,|−⟩ where $|\pm \rangle =(|0\rangle \pm |1\rangle )/\sqrt{2}$, transmitting this sequence to receiver Bob. Potential eavesdropper Eve intercepting this sequence must randomly choose measurement bases |0⟩,|1⟩ or |+⟩,|−⟩. Erroneous basis selection disturbs quantum states, inducing detectable error rates during Alice-Bob verification. This mechanism enables communication termination upon eavesdropping detection, thereby establishing protocol security.

Integrating methodologies from references [19,20,26,59], this work achieves controlled bidirectional quantum teleportation (CBT) of low-dimensional single-particle states via high-dimensional entangled channels. Principal contributions include:

(i) Construction of (d×f)-dimensional non-symmetric bases via single-body unitary operations, subsuming (3×2)-dimensional and (d×2)-dimensional cases. We also construct 3-dimensional and *d*-dimensional maximally entangled 5-particle states by using the high-dimensional Hadamard gate and controlled NOT gate. Our two types of constructions are key to implementing CBT schemes between two parts of arbitrary different dimensions.

(ii) By making use of a three-dimensional maximally entangled 5-qutrit state as quantum channel and hiring asymmetric base measurement, we propose a scheme to exchange two 2-dimensional unknown single-qubit states under the control of a supervisor, give general analytical expressions for the measurement outcomes, the corresponding collapsed states and the recovery operations, and calculate that the overall probability of success of the scheme is 100%.

(iii) By substituting the quantum channel in (ii) by a three-dimensional non-maximally entangled 5-qutrit state, the above unknown quantum states is exchanged simultaneously in such a way that they can be probabilistically restored through introducing auxiliary qubits and performing appropriate operations by the receivers after the sender performs non-symmetric basis measurements, where the probability of success is provided. The analysis shows that this scheme is a generalization of that in (ii).

(iv) We generalize the above two schemes from two aspects: (a) by substituting the quantum channels in (ii) and (iii) with two *d*-dimensional maximally entangled and non-maximally entangled 5-qudit states, respectively. (b) Replace the transmitted 2-dimensional unknown single-qubit states in (a) with two *f*-dimensional sing-particle states. Of course, the general analytical expressions of the sender’s measurement, the corresponding collapsed state, the receiver’s recovery operation, and the success probability of the schemes are given, respectively. This highly inductive generalization of mathematics not only greatly simplifies the originally lengthy description, but also reveals the inherent connections between a series of local unitary operations implemented by participants.

This work’s significance extends beyond the proposed protocols, opening avenues for advanced quantum teleportation research. It prompts broader considerations regarding feasibility and scalability, while raising fundamental challenges: teleportation of high-dimensional entangled states or multi-qubit systems via higher-dimensional channels presents compelling directions for future investigation. Recent work by Taufiqi et al. [62] has demonstrated that deterministic teleportation can be achieved even with non-maximally entangled five-qubit states, suggesting potential extensions of our framework.
